# Widespread Dysregulation of MiRNAs by *MYCN* Amplification and Chromosomal Imbalances in Neuroblastoma: Association of miRNA Expression with Survival

**DOI:** 10.1371/journal.pone.0007850

**Published:** 2009-11-16

**Authors:** Isabella Bray, Kenneth Bryan, Suzanne Prenter, Patrick G. Buckley, Niamh H. Foley, Derek M. Murphy, Leah Alcock, Pieter Mestdagh, Jo Vandesompele, Frank Speleman, Wendy B. London, Patrick W. McGrady, Desmond G. Higgins, Anne O'Meara, Maureen O'Sullivan, Raymond L. Stallings

**Affiliations:** 1 Department of Cancer Genetics, Royal College of Surgeons in Ireland, Dublin, Ireland; 2 Children's Research Centre, Our Lady's Children's Hospital Crumlin, Dublin, Ireland; 3 Center for Medical Genetics, Ghent University Hospital, Ghent, Belgium; 4 Children's Oncology Group Statistics and Data Center, University of Florida, Gainesville, Florida, United States of America; 5 Conway Institute of Biomolecular and Biomedical Research, University College Dublin, Dublin, Ireland; 6 Departments of Oncology and Pathology, Our Lady's Children's Hospital Crumlin, Dublin, Ireland; Institute of Cancer Research, United Kingdom

## Abstract

MiRNAs regulate gene expression at a post-transcriptional level and their dysregulation can play major roles in the pathogenesis of many different forms of cancer, including neuroblastoma, an often fatal paediatric cancer originating from precursor cells of the sympathetic nervous system. We have analyzed a set of neuroblastoma (n = 145) that is broadly representative of the genetic subtypes of this disease for miRNA expression (430 loci by stem-loop RT qPCR) and for DNA copy number alterations (array CGH) to assess miRNA involvement in disease pathogenesis. The tumors were stratified and then randomly split into a training set (n = 96) and a validation set (n = 49) for data analysis. Thirty-seven miRNAs were significantly over- or under-expressed in *MYCN* amplified tumors relative to *MYCN* single copy tumors, indicating a potential role for the MYCN transcription factor in either the direct or indirect dysregulation of these loci. In addition, we also determined that there was a highly significant correlation between miRNA expression levels and DNA copy number, indicating a role for large-scale genomic imbalances in the dysregulation of miRNA expression. In order to directly assess whether miRNA expression was predictive of clinical outcome, we used the Random Forest classifier to identify miRNAs that were most significantly associated with poor overall patient survival and developed a 15 miRNA signature that was predictive of overall survival with 72.7% sensitivity and 86.5% specificity in the validation set of tumors. We conclude that there is widespread dysregulation of miRNA expression in neuroblastoma tumors caused by both over-expression of the *MYCN* transcription factor and by large-scale chromosomal imbalances. MiRNA expression patterns are also predicative of clinical outcome, highlighting the potential for miRNA mediated diagnostics and therapeutics.

## Introduction

Neuroblastoma, one of the most common solid tumours in children, accounts for 15% of childhood cancer deaths [1]. Heterogeneity in the clinical behaviour of these tumors is remarkable, ranging from spontaneous regression to aggressive clinical course and death due to disease. Many biological factors such as patient age, ploidy, disease stage and histopathological characteristics are partially predictive of clinical outcome. Tumours in infants that are characterized by hyperdiploidy have a high propensity to spontaneously regress or differentiate into benign ganglioneuroma, even when the disease is widely disseminated. In contrast, tumours from children over 18 months of age are usually metastatic at diagnosis, often become refractory to treatment, and exhibit many recurrent chromosomal imbalances and/or amplification of the *MYCN* oncogene.

Amplification of the *MYCN* transcription factor was one of the first genetic abnormalities to be associated with poor clinical outcome in neuroblastoma [2]. A number of large scale chromosomal imbalances, including loss of either 1p or 11q or gain of 17q, are also associated with poor survival [3]–[5]. *MYCN* amplification (MNA) and loss of 11q are inversely related, defining independent genetic subtypes of the disease, with loss of chromosome 1p occurring preferentially in MNA tumors, and loss of 3p with 11q- tumors [6], [7].

The deregulation of microRNAs (miRNA) has recently been implicated in the pathogenesis of neuroblastoma. MiRNAs regulate gene expression at a post-transcriptional level by inhibiting mRNAs from being translated or causing them to be degraded. They play major roles in the differentiation of neural cells [8], and the deregulation of these sequences can contribute to tumorigenesis in many forms of cancer (see [9] for review). In neuroblastoma, studies have demonstrated that miR-34a, mapping to a region on chromosome 1p that is often deleted in high stage disease, acts as a tumor suppressor [10]–[12], as ectopic over-expression of this miRNA decreases cell proliferation through the induction of apoptosis. In addition, some miRNAs, such as miR-17-5p, behave in an oncogenic manner in these tumors, being directly up-regulated by MYCN [13].

Expression profiling of miRNAs in neuroblastomas has identified a number of miRNAs that are differentially expressed in favourable versus unfavourable tumor subtypes, particularly with respect to MNA versus non-MNA tumors [13]–[15]. However, these studies have been limited with respect to the number of tumors examined, with the largest study involving only 35 tumors profiled for 150 miRNAs [14]. Direct association of miRNA expression with patient survival could not be accomplished in such small data sets. Here, we expression profile 430 miRNA loci in 145 tumors that have also been characterized by high resolution aCGH, allowing identification of miRNAs which are associated with patient survival, differentially expressed in genetic subtypes, and that have been dysregulated by both over-expression of the MYCN transcription factor and large-scale chromosomal imbalances.

## Results

### Analysis of Somatically Acquired DNA Copy Number Alterations

High resolution aCGH analysis was carried out on all 145 primary neuroblastoma tumours in order to identify DNA copy number alterations and to classify the tumors into major genetic subtypes (*MYCN* amplified, chromosome 11q loss etc). Thirty-six tumors had MYCN amplification (MNA), four of which also had large-scale hemizygous loss of chromosome 11q material. In total, 42 tumors exhibited loss of chromosome 11q material without MNA. Many additional recurrent genomic imbalances, such as loss of 1p, 3p and gain of 17q, were also detected. For tumors that did not have MNA or 11q loss, 22 were derived from patients with stage 4 disease, while 45 came from patients with stage 1, 2, 3 or 4s disease.

### A miRNA Expression Profile Associated with MYCN Amplified Tumors

Of the 430 miRNAs profiled in this study, 132 were expressed in fewer than 10 tumors, so that the final dataset comprised 298 miRNAs expressed in at least 10 samples (Table S1).

**Figure 1:**
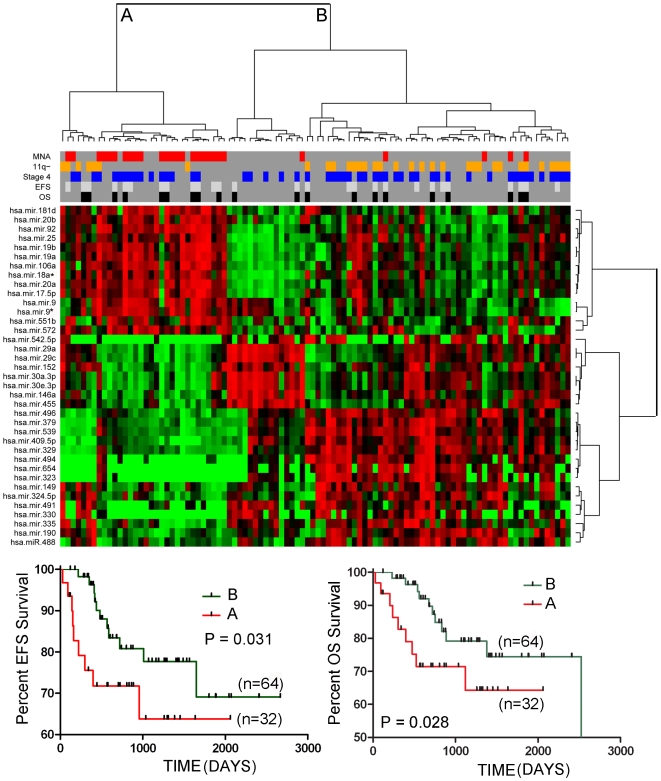
Two-way hierarchical cluster analysis on the training set of tumors (n = 96) using 37 miRNAs that were significantly differentially expressed between MNA and non-MNA tumors. Tumor cluster A was significantly enriched for MNA tumors (69%). The Kaplan-Meier graphs at the bottom of the figure show that EFS and OS was significantly reduced in the MNA tumor cluster (Cluster A) relative to the non-MNA cluster (Cluster B).

Given the importance of MYCN amplification in neuroblastoma, we initially identified miRNAs that were differentially expressed between MNA versus non-MNA tumors using the Wilcoxon Rank test based p-values corrected for multiple comparisons (using the training set of tumors, n = 96). A total of 37 miRNAs were significantly differentially expressed (p<0.05) with respect to MNA and are likely directly or indirectly regulated by MYCN. Fourteen miRNAs were over-expressed while 23 were under-expressed, as illustrated in Figure 1. Excluding members of the same polycistronic cluster, or major versus minor forms of the same miRNA locus, there were 8 over-expressed and 19 under-expressed independent miRNA transcriptional units.

**Figure 2:**
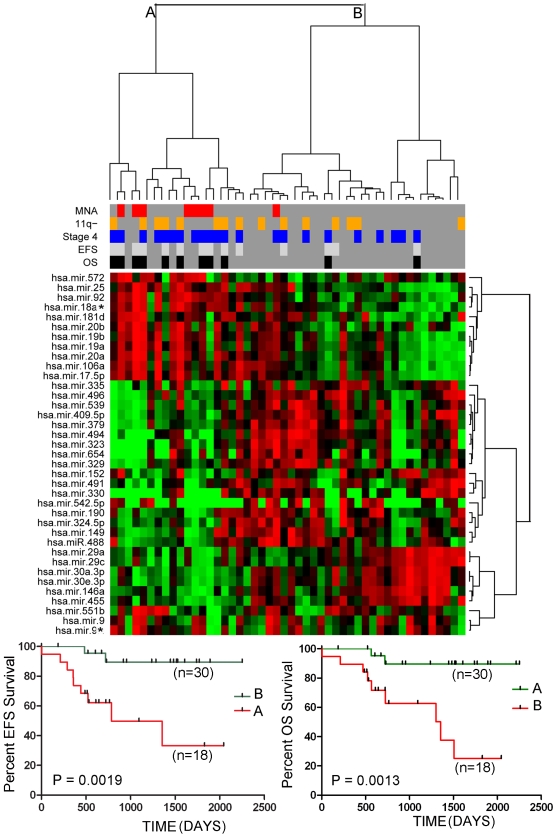
Two-way hierarchical cluster analysis on the validation set of tumors (n = 48) using 37 miRNAs that were significantly differentially expressed between MNA and non-MNA tumors. Tumor cluster A was significantly enriched for stage 4 tumors with MNA tumors. The Kaplan-Meier graphs at the bottom of the figure show that EFS and OS was significantly reduced in the MNA tumor cluster (Cluster A) relative to the non-MNA cluster (Cluster B).

As one would expect, two-way hierarchical cluster analysis with this set of miRNAs showed significant clustering of MNA tumors in both the training set (Figure 1) and the validation set of tumors (Figure 2). Six MNA tumors were misclassified, and a number of non-MNA tumors clustered within the major MNA clusters. The patients from the cluster (A) enriched for MNA tumors had significantly poorer event free survival (EFS) and overall survival (OS) than did the non-MNA cluster (B) in both the training and validation tumor sets (P values ranging from 0.0013 to 0.031). Analysis of survival of patients falling into smaller subclusters did not yield significant P values.

Two major miRNA expression clusters were discernable following cluster analysis with the MNA associated miRNA signature (Figure 1). One cluster was over-expressed in MNA tumors, while the other was under-expressed. Among the miRNAs over-expressed in MNA tumors was the polycistronic cluster miR-17-5p-92. This cluster has been previously reported to be highly expressed in MNA tumors [13]–[15].

In order to test the possibility that MYCN is either directly or indirectly regulating the expression of miRNA loci in an experimental system, TLDA microfluidic cards (368 miRNAs) were used to profile the *MYCN* inducible cell line, SHEP-TET21. SHEP-TET21 is a ‘tet-off’ cell line, only expressing *MYCN* in the absence of doxycycline (Figure S1). Using a fold change cut-off of ≤−1.5 or ≥+1.5, twenty-nine miRNAs were differentially expressed in *MYCN* ‘on’ compared to *MYCN* ‘off’ cells (Table S2). Nine out of these 29 miRNAs (31.0%) also showed differential expression in *MNA* versus non-MNA tumors. The direction of miRNA expressional change was identical in SHEP cells and tumors. It should be emphasized that these experiments only provide a correlation between MYCN expression change and miRNA expressional alterations, and that further experiments involving chromatin immunoprecipitation are required to distinguish between the direct and indirect effects of this transcription factor. Many reasons exist as to why the cell line repressible system had only 31% overlap with the tumors. For example, there could be very substantial differences in the promoter regions of the miRNA loci in the SHEP cell line versus the tumors, as well as differences in the expression of other transcription factors/repressors.

### Identification of a miRNA Expression Signature Predictive of Clinical Outcome

**Figure 3:**
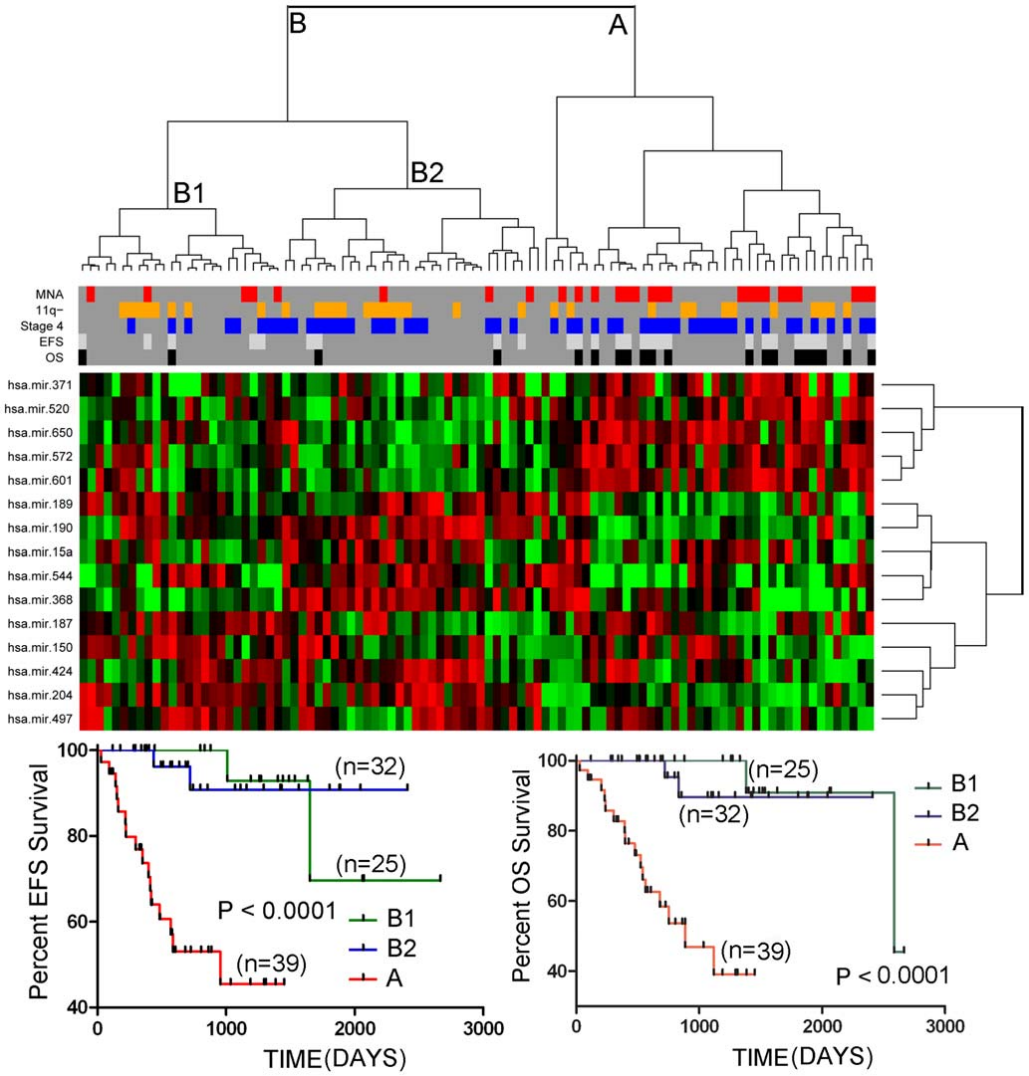
Two-way hierarchical cluster analysis on the training set of tumors (n = 96) using the top 15 miRNAs that were significantly associated with OS, as determined by the Random Forest classifier. As illustrated by the Kaplan-Meier graphs at the bottom of the figure, the patients from the two major cluster (A and B) differed dramatically in both EFS and OS. Cluster A is highly enriched for stage 4 tumors with MNA or loss of 11q and for patients with EFS and OS events. Notably, subclusters (B1 and B2) within the major cluster B showed a minor, but statistically significant difference in both EFS and OS.

**Figure 4:**
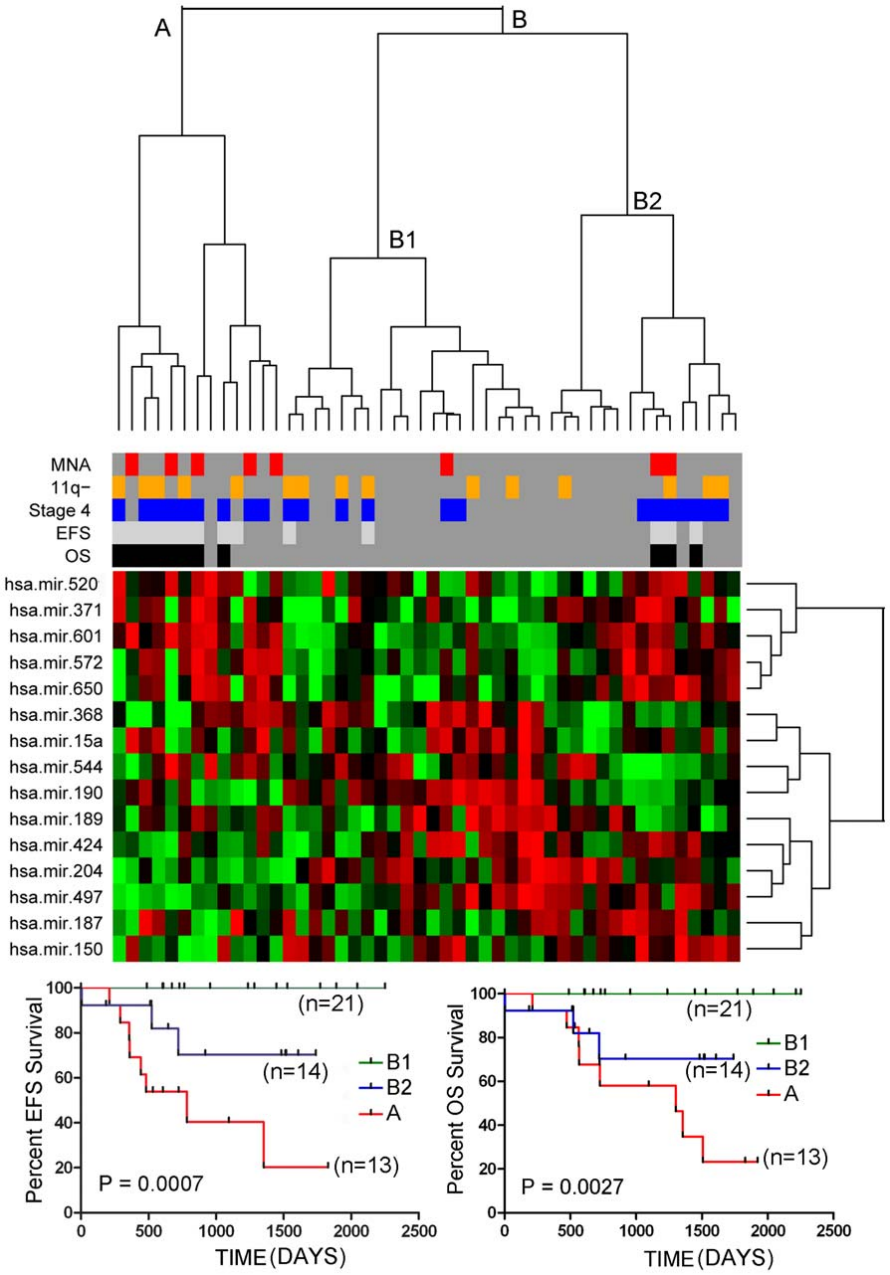
Two-way hierarchical cluster analysis on the validation set of tumors (n = 48) using the top 15 miRNAs that were significantly associated with OS, as determined by the Random Forest classifier. Similar to the training set of tumors, cluster A is highly enriched for stage 4 tumors with MNA or loss of 11q and for patients with EFS and OS events. As illustrated by the Kaplan-Meier graphs at the bottom of the figure, the patients from the two major clusters (A and B) differed dramatically in both EFS and OS. Notably, subclusters (B1 and B2) within the major cluster B showed a minor, but statistically significant difference in both EFS and OS.

Although the 37 miRNAs associated with *MYCN* expression levels was somewhat predictive of clinical outcome, we reasoned that a better signature could be derived by identifying miRNAs that are specifically associated with survival, given that many of the miRNAs in the *MYCN* signature might be only passively involved with tumorigenesis, and because other genetic subtypes, such as those with 11q loss, are also associated with poor survival. We used the Random Forest classifier to identify the top 15 miRNAs whose expression was most significantly associated with patient overall survival from the training set of tumors. Hierarchical cluster analysis with the top 15 miRNAs from this list produced two major clusters (A and B) which differed significantly in survival in both the training (p<0.0001) (Figure 3) and validation tumor sets (p<0.002)(Figure 4). Cluster B could be further split into two subclusters (B1 and B2) with minor, although statistically significant differences in survival characteristics (Figure 3 and 4). For overall survival in the validation tumor set, this miRNA expression signature had a sensitivity of 72.7%, a specificity of 86.5%, a positive predictive value of 61.5% and a negative predictive value of 91.4%. Two miRNAs, miR-190 and miR-572, in the miRNA survival signature were also significantly differentially expressed in MNA tumors versus non-MNA tumors.

### MiRNA Expression Is Dysregulated by DNA Copy Number Alterations

**Figure 5:**
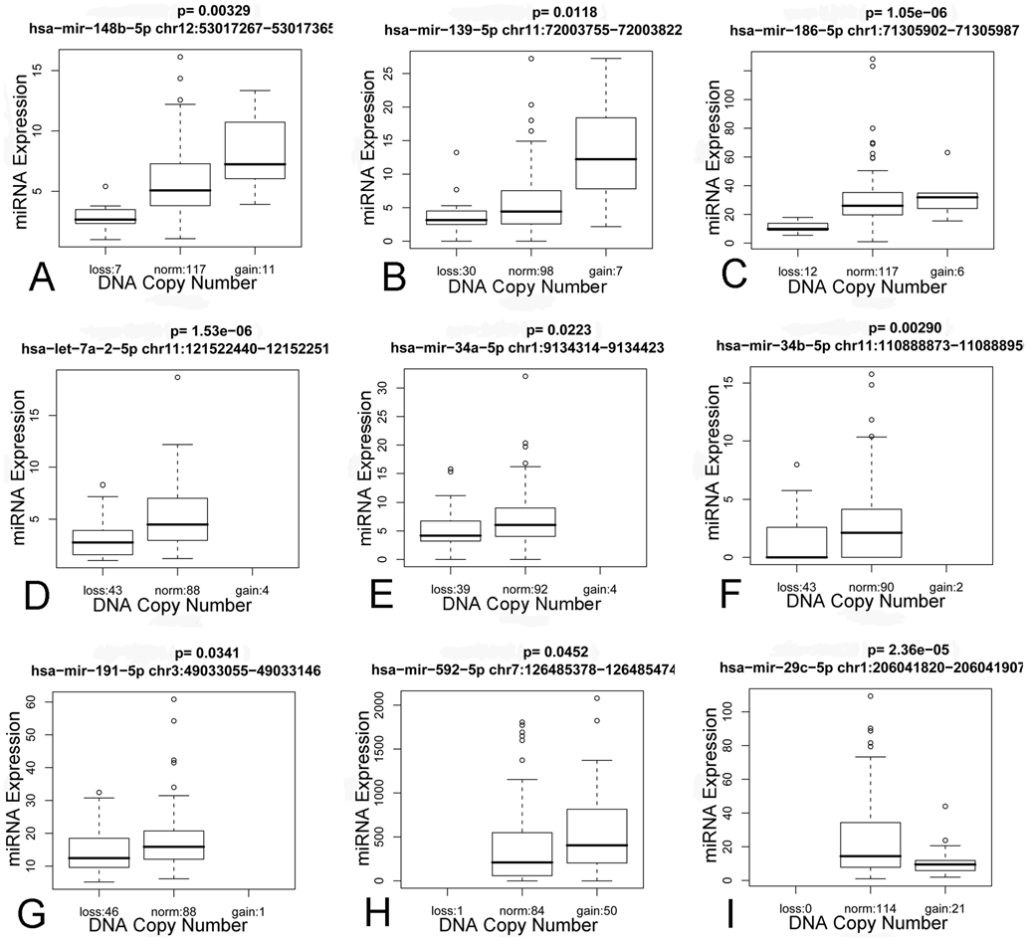
Representative box-plots illustrating correlation between miRNA expression and DNA copy number status. MiRNA locus, map location and p-value are displayed at the top of each box-plot while number of tumors having deletion, gain or normal DNA copy number status are displayed at the bottom. A through C are examples of miRNA expressional changes for regions that were both lost and gained in tumors. D through G are miRNA expressional changes for regions that showed only loss in the tumors, while H is an example of a miRNA mapping to a region that only showed gain in the entire tumor cohort. In the examples A through I miRNA expressional change is concordant with DNA copy number status, while box-plot I shows an example (miR-29c) of discordance between expression and copy number (i.e. tumors showing DNA copy number gain had significantly lower expression).

In order to determine if large-scale genomic gains and losses might have widely impacted upon miRNA expression, we correlated gains and losses of genomic regions, as determined by aCGH on each tumor, with relative miRNA expression values. Only miRNAs expressed in a minimum of 10 tumors and mapping to regions exhibiting loss or gain in a minimum of 5 tumors were included in our analysis. A total of 51 miRNA loci exhibited significant (P<0.05) differences in mean expression between tumors exhibiting a copy number alteration versus those with diploid status (Table S3 and Figure 5A–I). Because some loci (Figure 5A to C) were deleted in some tumors, and gained in others, there were a total of 62 genomic events affecting these 51 miRNA. In 47 instances, the alterations in miRNA expression were concordant with the DNA copy number change, e.g. lower expression for deletions, higher expression for DNA copy number gains. Although there were 15 instances where miRNA expressional alterations were inversely related to the genomic imbalance, the skewing towards concordant events (47 to 15) was highly non-random (p = 0.0002), leading us to conclude that genomic imbalances have significantly altered the expression levels of approximately 15% of all miRNA loci used in our expression profiling study.

## Discussion

Our results indicate that amplification of the MYCN transcription factor has significantly affected miRNA expression in neuroblastoma tumors, consistent with our earlier observations [14] and the observations of other groups [13], [15]. Our present studies, based on a larger set of miRNAs and tumors than what was previously studied, has permitted us to identify many additional miRNAs whose expression levels are correlated with MYCN levels. In addition, we demonstrate that many of the recurrent large-scale chromosomal imbalances, including loss of 1p, 3p, 11q and 14q, along with gain of 1q and 17q, have also had a major impact upon miRNA expression. Our finding that approximately 15% of miRNA loci have expressional changes attributable to genomic imbalances is comparable to the results of Zhang et al [16] showing that 15% of miRNAs are deregulated by such alterations in ovarian cancer. Interestingly, we identified several miRNAs whose expression levels were inversely correlated to the genomic imbalances, indicating that other factors can significantly negate the effects of DNA copy number alteration. For example, miR-29c is expressed at lower levels in MNA tumors in spite of the fact that it maps to a region on chromosome 1q that is commonly gained in neuroblastoma. In this instance, and in at least two additional instances (miR-30a and miR-25), over-expression of the *MYCN* oncogene would appear to be the factor that is over-riding the effect of DNA copy number change.

Many of the miRNAs that are down or up-regulated by either MYCN or chromosomal imbalances are likely to play key roles in tumorigenesis. For example, miR-34a mapping to chromosome 1p is clearly down-regulated by 1p deletion because it targets important pro-proliferation genes [11], [12], while the miR-17-5p cluster is directly up-regulated by MYCN and targets the p21 tumor suppressor. Wei et al [17] showed that over-expression of the miR-17-5p-92 cluster host gene, *MIRHG1*, is correlated with poor patient survival and that expression of miR17-5p-92 is positively correlated with host gene expression. Presumably over-expression of miR-17-5p-92 is also correlated with poor survival, although this was not directly reported on. Our results indicate a correlation of over-expression of the miR17-5p-92 cluster members with poor patient survival (p<0.009), although the p-value was not significant following a highly conservative correction for multiple comparisons. In this regard, the lack of a significant p-value associated with miR-17-5p over-expression and poor survival is probably a false negative result in our study, particularly in view of the fact that functional studies of this miRNA in neuroblastoma have indicated an oncogenic role in neuroblastoma [13].

Interestingly, miR-29a, which we show to be under-expressed in MNA tumors, was also recently shown to be down-regulated in neuroblastoma as it directly targets B7-H3, a cell surface immunomodulatory glycoprotein which contributes to the process of immune escape [18]. It is equally important to realize that some of the dysregulated miRNAs might be passive events and have no effect on disease pathogenesis. For example, miR-30e was highly under-expressed in MNA tumors, but we have previously shown that ectopic over-expression of this miRNA in MNA cell lines has no detectable impact on cell proliferation [12].

We have also demonstrated that a signature based on the expression of 37 miRNAs that are differentially expressed in MNA tumors relative to non-MNA tumors permits the clustering of patients into two groups that differ significantly in survival. However, this signature yields P-values that are not as good as the use of MNA or 11q status by themselves, possibly because some of the miRNAs in the signature play no role in tumorigenisis (e.g. miR-30e). The inclusion of these passive miRNAs in the MYCN miRNA signature could decrease the predicting power of a miRNA expression signature based on miRNAs identified by their association with MYCN levels. We therefore developed a miRNA expression signature by directly identifying the miRNAs that were most significantly associated with overall survival. Interestingly, only miR-572 and miR-190 from the set of 15 miRNAs most closely associated with EFS and OS overlapped the set of 37 miRNAs that were associated with MYCN amplification. This signature classified patients into groups and subgroups associated with EFS and OS with p-values that were significantly better than those obtained with the *MYCN* associated miRNA signature. This 15 miRNA expression signature requires validation in an independent tumor set, and represents a method of patient stratification that is independent of *MYCN* amplification status.

Under-expression of miRNAs in MNA tumors appears to occur twice as frequently as over-expression. This observation is consistent with our studies on a smaller set of miRNAs (n = 150)[14] and with observations on a related family member, c-MYC [19]. One of the notable miRNAs under-expressed in MNA tumors was the large polycistronic miRNA 379–656 cluster; a 44.7 kb polycistron containing 38 miRNAs mapping to chromosome 14. Four miRNAs from this cluster were expressed at significantly lower levels in MNA tumors relative to non-MNA tumors. Gene ontology analysis of predicted gene targets for miRNAs from this cluster revealed significant over-representation of biological processes such as neurogenesis, embryonic development and transcriptional regulation [20].

Treatment regimens for patients with neuroblastoma categorized as high risk involve intensive, multi-modal chemotherapy. Despite the severity of treatment, in many cases they have only short-term effects, emphasizing the fact that accurate identification of low and high risk is of great importance. The identification of clinical relevant miRNAs is an initial step towards identifying miRNAs as significant prognostic markers in neuroblastoma with potential novel therapeutic applications for this disease.

## Materials and Methods

### Cell Lines and Reagents

The MYCN-inducible cell line, SHEP-TET21, was obtained from Dr. Louis Chesler with permission of Prof. Manfred Schwab. RNA was isolated from this cell line 72 hrs following the addition of doxycline.

### Patient Cohort

Tumor material was obtained from either the Children's Oncology Group Tumour Bank (n = 108), or the Tumour Bank at Our Lady's Children's Hospital, Dublin (n = 37). Patients were treated under either the U.S. neuroblastoma treatment protocol or the European treatment protocol between 1998 to 2004. This work was approved by the Research Ethics Committee of the Royal College of Surgeons on 16th October 2007 (application No. REC241) and by the Research Ethics Committee of Our Lady's Children's Hospital on 5th August 2008 (application number GEN/70/07). The tumour cohort came from patients with stage 4 (n = 74), stage 3 (n = 31) and stages 1, 2 and 4s (n = 40) disease (INSS). In total, 36 tumors had MYCN amplification (MNA) while 42 tumors had loss of 11q without MNA (Table S4). *MYCN* amplification and loss of 11q were predictive of both poor EFS (p<0.0001) and OS (p<0.0001) in this set of 145 tumors (Figure S2).

### miRNA Extraction

Total RNA isolation was preformed using miRNeasy (Qiagen), as per manufacturers instructions.

### qPCR Using Gene Expression Assays

Reverse transcription was carried out with a TaqMan Reverse Transcription kit using random primers (Applied Biosystems). PCR amplification reactions contained TaqMan Fast Master Mix, cDNA and TaqMan probe & primers specific to *MYCN* and RPLPO ribosomal protein (Applied Biosystems). Relative quantification of gene expression was determined using the comparative cycle threshold method (2^−ΔΔCT^).

### Western Blotting

Denaturing polyacrylamide gel electrophoresis (SDS-PAGE) followed by immunoblotting for the detection of the antigen recognised by MYCN antibody (Abcam) were performed by standard methods.

### miRNA Reverse Transcription

RNA was reverse transcribed using the miRNA reverse transcription kit (Applied Biosystems). Cell line RNA was used in combination with the stem-loop Multiplex primer pools (Applied Biosystems), allowing reverse transcription of 48 different miRNA in each of 8 RT pools. The Megaplex primer pool (Applied Biosystems) was used with RNA from tumor samples, allowing simultaneous reverse transcription of 430 miRNAs and 36 endogenous controls in one RT pool [21].

### Pre-Amplification of cDNA from Clinical Samples

Megaplex RT product (5 µl) was pre-amplified using Applied Biosystems' TaqMan PreAmp Master Mix (2×) and PreAmp Primer Mix (5×). The PreAmp primer pool contained forward primers specific for each miRNA, and a universal reverse primer (Applied Biosystems, early access).

### Real-Time qPCR

#### Cell lines

Each cell line sample was profiled using TaqMan Low Density Arrays (TLDA) (368TaqMan PCR assays)(Applied Biosystems).

#### Tumor samples

For each cDNA sample, 430 miRNAs plus 36 control small RNAs were profiled using individual TaqMan PCR assays setup in 384-well format. As instrument and liquid handling variations were shown to be minimal, no PCR replicates were measured [21].

A Ct value of 35 represents single molecule template detection, and therefore a Ct value greater than 35 was considered noise. All miRNAs with Ct values greater than 35 were considered ‘non amplified’ or ‘not expressed’.

Prior to calculating relative expression values, mean normalization was carried out by subtracting the mean sample Ct from the individual miRNA Ct values [22]. Normalized relative expression (NRQ) of miRNA was calculated with reference to the Ct max (maximum Ct value for an individual miRNA across all tumour samples) using: NRQ = 2^(Ctmax−Ct)^.

### Data Analysis

Micro-RNA profiles for 145 tumors were used in this study. To investigate which microRNAs varied over the various sample classes of MNA and non-MNA and Survival and non-Survival we split these into a *training/test set* (66%) and a *hold-out* set (33%). This latter hold-out data set plays no part in training or testing and is used to independently validate of findings. The selection was stratified in that approximately the same rate of deaths occurred in both the training/test and hold-out sets.

#### Significance testing

The significance of miRNA differential expression over tumor sub-types was evaluated by assigning P-values based on the non-parametric Wilcoxon rank sum test. Adjustment for multiple comparisons was made using the Benjamini-Hochberg multiple testing correction. The resultant set of miRNAs were then examined using hierarchical cluster analysis and the principal split was found, as expected, to occur between MNA and non-amplified tumors. A similar investigation into overall survival (OS) and event free survival (EFS) was also carried out. In both cases the hold-out set was used to examine the generalizability of the models.

#### The Random Forest and feature selection

Random Forest [23] is an Ensemble Classifier in which the base classifier is an un-pruned *Decision Tree* built from a random selection of feature variables (in this case miRNAs) for a randomly selected subset of training samples (tumors). The association or *importance* of a feature with a particular class may be by garnered by assessing its impact on classification. This is achieved by randomizing the feature values over sample classes and re-running the classifier. If there is a large adverse affect upon the classification the feature achieves a high importance score. The advantage of an *importance score* over simple filtering methods (p-value, information gain etc) is that multivariate feature interaction effects may be assessed. An additional advantage of Random Forest is its reduced tendency to *overfit* a dataset i.e. when a method models noise within a dataset and not the phenomenon under investigation. Lastly Random Forest may be considered a non-parametric method as information gain is used to build the base decision tree classifiers. The feature *importance score* derived from the Random Forest classifier was used to assess the association of a particular set of miRNAs with survival. This signature was first developed on the training/test set of tumors and the hold-out set was used as an external validation set.

#### Cluster analysis and visualization

Hierarchical clustering and heatmap generation was implemented using the *hclustp* and *heatmap.2* functions of the R statistical computing language v2.8.0.

#### Statistical analysis of neuroblastoma primary tumors

EFS time was calculated from the time of enrolment on the front-line or biologic study until the time of the first occurrence of relapse, progressive disease, secondary malignancy, or death, or until the time of last contact if no event occurred. OS time was calculated until the time of death or until last contact. EFS and OS are presented as the estimate +/− the standard error.

#### Array CGH

Array CGH was carried out as previously described [24] using a 72,000 feature array (NimbleGen).

## Supporting Information

Figure S1 Western blot shows major differences in MYCN protein levels in: lane 1: a MYCN amplified NBL cell line, Kelly, lane 2: SHEP-TET21 cells in the absence of doxycycline, lane 3: SHEP-TET21 cells in the presence of doxycycline.
(2.88 MB TIF)

Figure S2 Kaplan-Meier plots demonstrating that MNA and 11q loss were both predictive of poor survival in the entire cohort of 145 tumors.
(0.16 MB TIF)

Table S1 List of miRNAs expressed in at least 10 tumors
(0.01 MB PDF)

Table S2 Differentially expressed miRNAs in SHEP treated (low MYCN levels) versus SHEP untreated (high MYCN levels)
(0.01 MB PDF)

Table S3 MiRNAs With Expression Levels Correlating to Genomic Imbalances
(0.05 MB PDF)

Table S4 Clinical and Genetic Characteristics of Tumor Cohort
(0.01 MB PDF)
