# Supplementary figures and images for: Widespread Dysregulation of MiRNAs by MYCN Amplification and Chromosomal Imbalances in Neuroblastoma: Association of miRNA Expression with Survival
Source: PLoS One. 2009 Nov 16;4(11):e7850. doi: 10.1371/journal.pone.0007850 (PMC2773120; doi:10.1371/journal.pone.0007850)

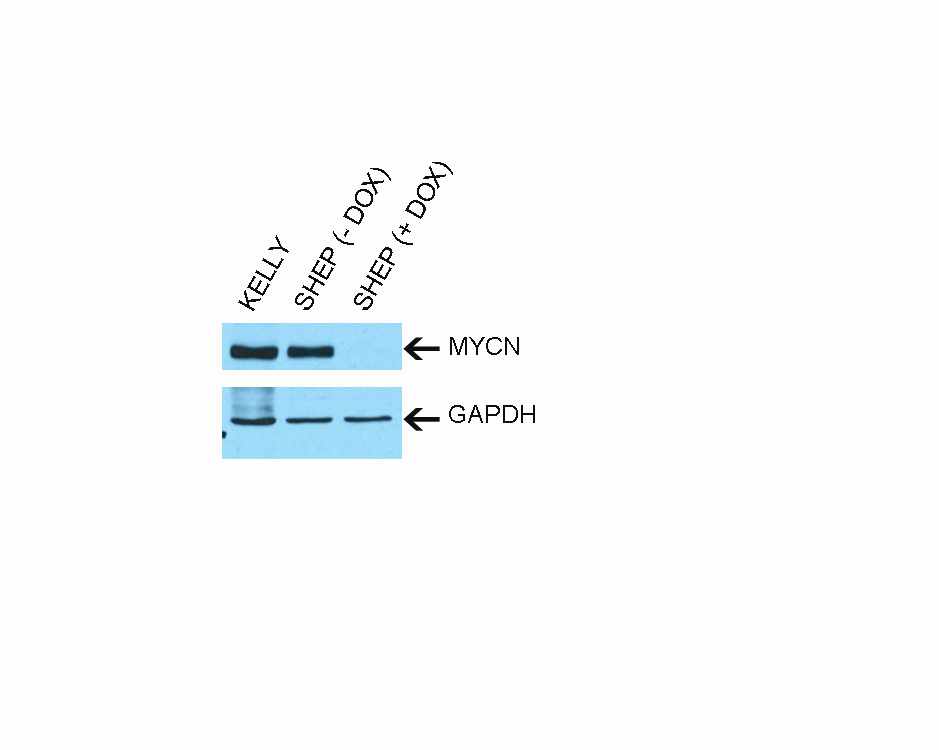

Supplement: Figure S1 — Western blot shows major differences in MYCN protein levels in: lane 1: a MYCN amplified NBL cell line, Kelly, lane 2: SHEP-TET21 cells in the absence of doxycycline, lane 3: SHEP-TET21 cells in the presence of doxycycline. (2.88 MB TIF) [file pone.0007850.s001.tif]
