# Supplementary material for: Widespread Dysregulation of MiRNAs by MYCN Amplification and Chromosomal Imbalances in Neuroblastoma: Association of miRNA Expression with Survival
Source: PLoS One. 2009 Nov 16;4(11):e7850. doi: 10.1371/journal.pone.0007850 (PMC2773120; doi:10.1371/journal.pone.0007850)
Supplement: Table S1 — List of miRNAs expressed in at least 10 tumors (0.01 MB PDF) [file pone.0007850.s003.pdf]

hsa-mir-139
hsa-mir-145
hsa-let-7b
hsa-mir-339
hsa-mir-500
hsa-mir-98
hsa-mir-132
hsa-mir-186
hsa-mir-329
hsa-mir-371
hsa-mir-133a
hsa-mir-224
hsa-mir-1
hsa-mir-551a
hsa-mir-200c
hsa-mir-181a
hsa-mir-103
hsa-mir-141
hsa-mir-346
hsa-mir-95
hsa-mir-30e-3p
hsa-mir-624
hsa-mir-30a-3p
hsa-mir-31
hsa-mir-449b
hsa-mir-28
hsa-mir-511
hsa-mir-199aH
hsa-mir-187
hsa-mir-320
hsa-mir-124a
hsa-mir-542-5p
hsa-mir-217
hsa-mir-376b
hsa-mir-508
hsa-mir-340
hsa-mir-106a
hsa-let-7f
hsa-mir-130b
hsa-mir-19b
hsa-let-7g
hsa-mir-189
hsa-mir-372
hsa-mir-326
hsa-mir-183
hsa-mir-330
hsa-mir-184
hsa-mir-125b
hsa-mir-99b
hsa-mir-485-5p
hsa-mir-153
hsa-mir-197
hsa-mir-520c
hsa-mir-433
hsa-mir-100

hsa-mir-24
hsa-mir-302b
hsa-mir-129
hsa-mir-299-5p
hsa-mir-15b
hsa-mir-135a
hsa-mir-22
hsa-mir-365
hsa-mir-520d
hsa-mir-204
hsa-mir-345
hsa-mir-218
hsa-mir-586
hsa-mir-19a
hsa-mir-29a
hsa-mir-381
hsa-mir-374
hsa-mir-331
hsa-mir-369-3p
hsa-mir-219
hsa-mir-148b
hsa-mir-565
hsa-mir-214
hsa-mir-146a
hsa-mir-33
hsa-mir-149
hsa-mir-369-5p
hsa-mir-515-5p
hsa-mir-25
hsa-mir-377
hsa-mir-30c
hsa-mir-199a
hsa-mir-27b
hsa-mir-101
hsa-mir-29b
hsa-mir-16
hsa-mir-524
hsa-mir-34c
hsa-mir-142-5p
hsa-let-7e
hsa-mir-627
hsa-mir-106b
hsa-mir-376a
hsa-mir-154
hsa-let-7i
hsa-mir-196a
hsa-mir-656
hsa-mir-518b
hsa-mir-601
hsa-mir-302aH
hsa-mir-126H
hsa-mir-337
hsa-mir-563
hsa-mir-361
hsa-mir-378

hsa-mir-432
hsa-mir-296
hsa-mir-128b
hsa-mir-375
hsa-mir-496
hsa-mir-143
hsa-let-7a
hsa-mir-93
hsa-mir-302a
hsa-mir-324-5p
hsa-mir-539
hsa-mir-92
hsa-mir-128a
hsa-mir-135b
hsa-mir-509
hsa-mir-505
hsa-mir-142-3p
hsa-mir-424
hsa-mir-452H
hsa-mir-206
hsa-mir-151
hsa-mir-23b
hsa-mir-155
hsa-mir-34b
hsa-mir-551b
hsa-mir-148a
hsa-mir-338
hsa-mir-15a
hsa-mir-10b
hsa-mir-203
hsa-mir-26b
hsa-mir-380-3p
hsa-mir-127
hsa-mir-21
hsa-mir-363
hsa-mir-18a
hsa-mir-660
hsa-mir-134
hsa-mir-630
hsa-mir-34a
hsa-mir-432H
hsa-mir-494
hsa-mir-190
hsa-mir-26a
hsa-mir-342
hsa-mir-335
hsa-mir-202H
hsa-mir-503
hsa-mir-193a
hsa-mir-493-3p
hsa-mir-30d
hsa-mir-7
hsa-mir-520g
hsa-mir-181c
hsa-mir-632

hsa-mir-449
hsa-mir-409-5p
hsa-mir-199b
hsa-mir-520f
hsa-mir-301
hsa-mir-497
hsa-mir-550
hsa-mir-192
hsa-mir-136
hsa-mir-10a
hsa-mir-422b
hsa-mir-544
hsa-mir-491
hsa-mir-99a
hsa-mir-423
hsa-mir-662
hsa-mir-17-5p
hsa-let-7d
hsa-mir-20b
hsa-mir-215
hsa-mir-191
hsa-mir-643
hsa-mir-517c
hsa-mir-542-3p
hsa-mir-216
hsa-mir-370
hsa-mir-152
hsa-mir-202
hsa-mir-367
hsa-mir-9H
hsa-mir-222
hsa-mir-410
hsa-mir-194
hsa-mir-210
hsa-mir-150
hsa-mir-422a
hsa-mir-302d
hsa-mir-501
hsa-mir-324-3p
hsa-mir-495
hsa-mir-133b
hsa-mir-32
hsa-mir-493
hsa-mir-30a-5p
hsa-mir-181d
hsa-mir-429
hsa-mir-548d
hsa-mir-30b
hsa-mir-196b
hsa-mir-502
hsa-mir-564
hsa-mir-650
hsa-mir-451
hsa-mir-328
hsa-mir-27a

hsa-mir-485-3p
hsa-mir-9
hsa-mir-17-3p
hsa-mir-302c
hsa-mir-125a
hsa-mir-221
hsa-mir-487b
hsa-mir-455
hsa-mir-188
hsa-mir-382
hsa-mir-489
hsa-mir-610
hsa-mir-137
hsa-mir-373
hsa-mir-572
hsa-mir-323
hsa-mir-574
hsa-mir-580
hsa-mir-147
hsa-mir-223
hsa-mir-654
hsa-mir-655
hsa-mir-20a
hsa-mir-604
hsa-mir-140
hsa-mir-200b
hsa-mir-411
hsa-mir-425
hsa-mir-516-3p
hsa-mir-526bH
hsa-mir-368
hsa-mir-616
hsa-let-7c
hsa-mir-18aH
hsa-mir-379
hsa-mir-639
hsa-mir-383
hsa-mir-651
hsa-mir-362
hsa-mir-487a
hsa-mir-29c
hsa-mir-486
hsa-mir-193b
hsa-mir-195
hsa-mir-130a
hsa-miR-185
hsa-miR-548c
hsa-miR-638
hsa-miR-380-5p
hsa-miR-504
hsa-miR-105
hsa-miR-488
hsa-miR-448
hsa-miR-126
hsa-miR-532

hsa-miR-594
hsa-miR-579
hsa-miR-628
hsa-miR-597
hsa-miR-548a
hsa-miR-545
hsa-miR-425-5p
hsa-miR-641
hsa-miR-617
hsa-miR-376aH
hsa-miR-146b
hsa-miR-592
hsa-miR-653
hsa-miR-606
hsa-miR-562
hsa-miR-589
hsa-miR-645
hsa-miR-642
hsa-miR-649
hsa-miR-629
hsa-miR-618
hsa-miR-576
hsa-miR-556
