# Supplementary material for: Widespread Dysregulation of MiRNAs by MYCN Amplification and Chromosomal Imbalances in Neuroblastoma: Association of miRNA Expression with Survival
Source: PLoS One. 2009 Nov 16;4(11):e7850. doi: 10.1371/journal.pone.0007850 (PMC2773120; doi:10.1371/journal.pone.0007850)
Supplement: Table S2 — Differentially expressed miRNAs in SHEP treated (low MYCN levels) versus SHEP untreated (high MYCN levels) (0.01 MB PDF) [file pone.0007850.s004.pdf]

MiRNA	Fold Change	Change in Tumor	Reported By Others
let-7d	2.5	X	*
miR-17-5p	5.6	X	*
miR-18a	3.4	X	
miR-19a	3.4	X	
miR-19b	3.6	X	
miR-20a	4.6	X	
miR-22	0.51		
miR-23b	0.54		
miR-25	2	X	
miR-28	0.69		
miR-92	3.3	X	*
miR-93	2		*
miR-98	0.56		
miR-99b	0.68		
miR-107	2.4		
miR-135b	0.59		
miR-140	0.65		
miR-142	0.36		
miR-143	3.4		
miR-189	0.46		
miR-335	0.39	X	
miR-342	1.6		
miR-378	5.7		
miR-500	2.2		
miR-501	1.9		
miR-565	1.6		
miR-576	1.9		
miR-615	0.57		
miR-660	2.2		

*Reported by Schulte et al
