# Supplementary material for: Widespread Dysregulation of MiRNAs by MYCN Amplification and Chromosomal Imbalances in Neuroblastoma: Association of miRNA Expression with Survival
Source: PLoS One. 2009 Nov 16;4(11):e7850. doi: 10.1371/journal.pone.0007850 (PMC2773120; doi:10.1371/journal.pone.0007850)
Supplement: Table S3 — MiRNAs With Expression Levels Correlating to Genomic Imbalances (0.05 MB PDF) [file pone.0007850.s005.pdf]

miRNA	Chromosome Location	No. of tumors with loss	MiRNA Expression[1]	P-value	No. of tumors with gains	MiRNA Expression[2]
mir-186	chr1:9134314−9134423	39	lower	p= 0.0223		
mir-29b	chr1:206042411−206042491			p= 0.00085	21	lower
mir-29c	chr1:206041820−206041907			p= 2.36e−05	21	lower
mir-30c−1−5p	chr1:40995543−40995631	29	lower	p= 0.00055		
mir-30e−3p	chr1:40992614−40992705	29	lower	p= 0.00916		
mir-34a−5p	chr1:9134314−9134423	39	lower	p= 0.0223		
let−7a−2−5p	chr11:121522440−12152251 10	43	lower	p= 1.53e−06		
mir−100−5p	chr11:121528147−12152822 6	43	lower	p= 0.000608		
mir−125b−1−5p	chr11:121475675−12147576	43	lower	p= 0.0101		
mir−139−5p	chr11:72003755−72003822	30	lower	p= 0.0118	7	higher
mir−192−5p	chr11:64415185−64415294	43	higher	p= 0.0445	8	higher
mir−34b−5p	chr11:110888873−11088895 6	43	lower	p= 0.00290		
mir−148b−5p	chr12:53017267−53017365	7	lower	p= 0.00329	11	higher
mir−26a−2−5p	chr12:56504659−56504742	5	lower	p= 0.0212	13	higher
mir−331−5p	chr12:94226327−94226420			p= 0.00203	19	higher
mir−616−5p	chr12:56199213−56199309	6	lower	p= 0.0471	12	higher
mir−17−3p	chr13:90800860−90800943			p= 0.00539	17	higher
mir−345−5p	chr14:99843949−99844046	30	lower	p= 0.0171		
mir−628−5p	chr15:53452430−53452524	8	lower	p= 0.00284		
mir−7−2−5p	chr15:86956060−86956169	7	higher	p= 0.0357	6	higher
mir−328−5p	chr16:65793725−65793799	31	lower	p= 0.0294		
mir−10a−5p	chr17:44012199−44012308			p= 0.00303	87	lower
mir−324−3p	chr17:7067340−7067422	16	lower	p= 0.000265	22	higher
mir−423−5p	chr17:25468223−25468316			p= 0.000806	32	higher
mir−548d−2−5p	chr17:62898067−62898163			p= 0.00138	99	higher
mir−632−5p	chr17:27701241−27701334			p= 0.0169	41	lower
mir−371−5p	chr19:58982741−58982807	50	lower	p= 0.00993		
mir−130b−5p	chr22:20337593−20337674	25	lower	p= 0.0182	5	higher
mir−15b−5p	chr3:161605070−161605167	11	lower	p= 1.05e−08		
mir−16−2−5p	chr3:161605227−161605307	11	lower	p= 0.0271		
mir−191−5p	chr3:49033055−49033146	46	lower	p= 0.0341		

mir−551b−5p	chr3:169752336−169752431	14	lower	p= 0.0413		
mir−563−5p	chr3:15890282−15890360	38	lower	p= 0.0305		
mir−302c−5p	chr4:113788968−113789035	10	higher	p= 0.0212		
mir-302d−5p	chr4:113788609−113788676	10	higher	p= 0.0176		
mir−572−5p	chr4:10979549−10979643	22	lower	p= 0.0104		
mir−218−2−5p	chr5:168127729−168127838			p= 0.0495	9	lower
mir−30a−3p	chr6:72169975−72170045			p= 5.36e−05	8	lower
mir−106b−5p	chr7:99529552−99529633			p= 0.0249	38	higher
mir−148a−5p	chr7:25956064−25956131			p= 0.00421	42	higher
mir−25−5p	chr7:99529119−99529202	5	higher	p= 0.0328	38	higher
mir−29a−5p	chr7:130212046−130212109			p= 0.00729	50	higher
29b−1−5p	chr7:130212758−130212838			p= 0.00963	50	higher
mir−335−5p	chr7:129923188−129923281			p= 0.0145	50	higher
mir−589−5p	chr7:5501976−5502074			p= 0.039	26	higher
mir−592−5p	chr7:126485378−126485474			p= 0.0452	50	higher
let−7f−1−5p	chr9:95978450−95978536			p= 0.0218	7	lower
mir−126−5p	chr9:138684875−138684959	39	higher	p= 0.0172	5	lower
mir−199b−5p	chr9:130046821−130046930	31	lower	p= 0.00876		
mir−219−2−5p	chr9:130194718−130194814	30	higher	p= 0.0111	6	higher
mir−491−5p	chr9:20706104−20706187	11	lower	p= 0.00105		
mir-186	chr1:9134314−9134423	39	lower	p= 0.0223		
mir−29b	chr1:206042411−206042491			p= 0.00085	21	lower
mir−29c	chr1:206041820−206041907			p= 2.36e−05	21	lower
mir−30c−1−5p	chr1:40995543−40995631	29	lower	p= 0.00055		
mir−30e−3p	chr1:40992614−40992705	29	lower	p= 0.00916		
mir−34a−5p	chr1:9134314−9134423	39	lower	p= 0.0223		
let−7a−2−5p	chr11:121522440−121522510	43	lower	p= 1.53e−06		
mir−100−5p	chr11:121528147−121528226	43	lower	p= 0.000608		
mir−125b−1−5p	chr11:121475675−12147576	43	lower	p= 0.0101		

Key:

[1]Median miRNA expression in tumors with DNA copy number loss is lower than in tumors without deletion.

[2]Median miRNA expression in tumors with DNA copy number gain is higher than in tumors without gain.

Median miRNA expression is discordant with DNA copy number alteration.
