# Supplementary material for: Widespread Dysregulation of MiRNAs by MYCN Amplification and Chromosomal Imbalances in Neuroblastoma: Association of miRNA Expression with Survival
Source: PLoS One. 2009 Nov 16;4(11):e7850. doi: 10.1371/journal.pone.0007850 (PMC2773120; doi:10.1371/journal.pone.0007850)
Supplement: Table S4 — Clinical and Genetic Characteristics of Tumor Cohort (0.01 MB PDF) [file pone.0007850.s006.pdf]

Tumor	1p-	MNA	3p-	11q-	17q+	INSS Stage	Age at Diagnosis	OS Event*
OLHC250	yes	no	no	no	yes	1	610	No
CHOP258	no	yes	yes	no	yes	4	2779	Death
CHOP1930	yes	no	no	yes	no	4	389	No
CHOP2291	no	no	no	no	no	2a	1631	No
CHOP1751	no	no	no	no	yes	2b	412	No
CHOP1551	yes	no	no	no	yes	4	4629	Death
CHOP2238	yes	yes	no	no	no	4	507	Death
OLHC456	yes	no	no	no	yes	4	277	No
CHOP1729	no	no	yes	yes	yes	4	2426	Death
CHOP2052	no	no	no	no	yes	3	785	No
CHOP_1389	no	no	no	yes	no	2b	598	No
OLHC312	yes	no	no	no	yes	2b	288	No
CHOP2221	no	no	yes	yes	yes	4	1961	No
CHOP155	no	no	no	no	yes	3	1701	No
OLHC48	no	no	no	yes	no	4	336	No
CHOP1821	no	no	no	no	yes	2a	256	No
OLHC460	yes	no	no	no	no	4	1121	No
CHOP2128	yes	no	no	no	yes	3	552	No
CHOP_269	no	no	no	no	no	2a	347	No
OLHC270	yes	yes	no	no	yes	4	329	No
CHOP1817	yes	yes	no	no	no	4	1010	No
CHOP211	no	no	no	no	yes	3	561	No
CHOP1357	no	no	no	no	no	3	628	No
CHOP1314	no	no	no	no	no	2a	1818	No
CHOP2243	no	no	no	no	yes	4	983	No
CHOP2045	no	no	no	yes	yes	4	1142	No
CHOP28	yes	yes	no	no	yes	3	606	Death
CHOP2158	no	no	yes	no	yes	3	918	No
CHOP_430	no	no	no	yes	no	4	160	Death
OLHC53	no	yes	no	no	no	2	1125	No
CHOP273	no	no	no	no	no	4	505	No
CHOP1040	yes	no	no	no	yes	2b	114	No
CHOP1281	no	no	no	no	yes	2a	2486	No
CHOP_136	no	no	no	yes	no	4	371	No
CHOP2315	no	no	no	no	no	2a	47	No
OLHC474	yes	no	no	yes	yes	4	2128	Death
CHOP_151	no	no	no	yes	no	2b	246	No
CHOP_1130	no	no	no	yes	no	1	4656	No
CHOP1396	no	no	no	no	no	3	2198	No
CHOP2172	yes	yes	no	no	yes	4	641	Death
OLHC417	yes	no	yes	yes	yes	4	2533	No
CHOP_355	no	no	no	yes	no	4	971	Death
CHOP1474	no	no	no	no	no	3	735	No
CHOP1710	yes	yes	no	yes	no	4	370	Death
OLHC277	yes	no	yes	yes	yes	3	1606	No
CHOP1957	no	no	no	no	yes	2a	95	No
CHOP1298	no	no	no	no	yes	2b	2717	No
CHOP2057	yes	no	no	no	no	4	559	Death
CHOP288	no	no	no	no	no	2a	72	No
CHOP1894	yes	yes	no	no	yes	4	211	Death
CHOP2200	yes	yes	no	no	yes	4	525	No
CHOP2119	yes	no	no	no	no	4	947	No
OLHC27	no	no	no	yes	no	2a	1095	No
CHOP1585	no	no	no	no	no	4	4608	Death

OLHC209	yes	yes	no	no	no	3	2011	No
CHOP1805	yes	no	yes	no	no	4	1928	No
CHOP1699	yes	no	no	no	yes	3	1659	Death
OLHC214	no	no	no	yes	yes	4	391	Death
CHOP265	no	no	no	no	yes	2b	435	No
CHOP1418	no	no	no	no	no	3	740	No
CHOP_296	no	no	no	yes	no	2a	826	No
CHOP_1195	no	no	no	yes	no	1	988	No
CHOP1643	yes	yes	no	no	no	4	473	Death
CHOP2285	yes	yes	no	no	yes	4	536	No
OLHC272	yes	yes	no	no	yes	4	1310	Death
CHOP2064	yes	no	no	no	no	4	975	No
CHOP2171	no	no	yes	yes	yes	4	1089	No
OLHC407	yes	no	yes	yes	yes	4	1383	No
CHOP1671	no	no	no	no	no	3	1029	No
CHOP2227	no	no	no	yes	yes	4	1366	No
CHOP1391	no	no	no	no	no	2b	583	No
CHOP1638	no	yes	yes	no	no	4	2259	Death
CHOP159	no	no	yes	no	yes	2a	202	No
OLHC255	no	yes	no	no	no	2	1314	No
OLHC394	yes	yes	yes	yes	yes	4	945	No
OLHC196	yes	no	yes	yes	yes	4s	704	No
OLHC455	yes	no	no	no	yes	4	128	No
CHOP1909	yes	no	no	no	no	4	547	No
OLHC276	yes	yes	no	no	yes	3	1007	No
CHOP1830	yes	yes	yes	yes	yes	4	3963	Death
CHOP1667	no	no	yes	yes	yes	3	1445	Death
CHOP2161	yes	no	no	yes	yes	4	1462	No
OLHC476	yes	no	yes	no	no	3	807	No
CHOP1853	no	no	yes	no	yes	4	4781	No
CHOP106	no	no	no	no	yes	2b	232	No
OLHC14	no	yes	no	no	no	4	751	Death
CHOP_178	no	no	no	yes	no	4	76	No
CHOP1992	yes	yes	no	no	yes	4	2008	No
CHOP2328	no	no	no	no	no	3	584	No
CHOP1473	yes	yes	no	no	yes	3	970	No
OLHC390	no	no	yes	no	yes	4	679	No
CHOP2173	yes	no	yes	yes	yes	4	1244	No
CHOP1915	yes	no	yes	yes	yes	4	2039	No
OLHC233	yes	no	yes	no	yes	3	175	No
CHOP300	yes	yes	no	no	yes	3	1112	Death
CHOP_1406	no	yes	no	no	no	2a	710	No
CHOP1427	no	no	yes	yes	yes	2b	698	No
CHOP_1200	no	no	no	yes	no	4	319	No
CHOP2148	yes	no	yes	yes	yes	4	1267	No
CHOP2142	no	no	yes	yes	yes	4	1330	No
CHOP_1012	no	no	no	yes	no	4	1843	No
OLHC283	yes	yes	no	no	yes	3	767	No
CHOP339	no	no	no	no	yes	2b	422	No
OLHC404	no	yes	no	no	yes	4	1066	Death
CHOP2239	no	no	no	yes	no	2a	781	No
CHOP1935	no	no	no	no	yes	4	1224	No
CHOP_1359	no	no	no	yes	no	4	1129	No
CHOP1096	no	no	no	no	no	3	706	No
OLHC195	yes	no	yes	yes	yes	4	2200	Death

CHOP1754	yes	no	yes	yes	yes	4	1406	No
CHOP1970	no	no	no	no	no	2a	460	No
CHOP1639	yes	yes	no	yes	yes	3	1250	No
CHOP1831	yes	yes	no	no	yes	3	559	No
OLHC36	no	no	no	no	no	2a	30	No
CHOP1652	yes	no	no	no	yes	2b	1610	No
CHOP347	yes	no	no	no	yes	2b	293	No
OLHC253	yes	yes	no	no	yes	3	588	No
CHOP1837	no	no	no	yes	yes	3	1011	No
CHOP1405	no	no	no	no	yes	4	699	No
OLHC459	yes	no	yes	yes	yes	4	1402	No
CHOP2120	yes	yes	no	no	yes	3	445	No
CHOP_1068	no	yes	no	no	no	4	405	Death
CHOP1851	yes	no	no	no	no	4	5238	Death
CHOP1064	no	no	no	no	no	3	1141	No
OLHC408	no	no	no	no	yes	4	139	No
CHOP_203	no	no	no	no	no	2b	156	No
CHOP_1173	no	no	no	yes	no	2b	1317	Death
CHOP81	no	no	no	yes	yes	3	1227	No
CHOP108	yes	yes	no	no	yes	3	877	No
CHOP2122	yes	no	no	no	no	4	1568	No
CHOP2165	yes	yes	no	no	yes	4	564	No
CHOP2174	yes	yes	no	no	yes	4	1514	No
OLHC217	no	no	no	no	no	3	44	No
OLHC401	no	no	no	no	yes	4s	58	No
CHOP2170	yes	no	yes	no	yes	4	1991	No
OLHC470	no	no	yes	yes	yes	4	1062	No
CHOP_493	no	yes	no	no	no	4	1500	Death
OLHC31	no	no	no	yes	no	4	691	Death
CHOP1960	no	no	no	no	no	2a	80	No
CHOP1651	no	no	no	no	no	4	789	No
CHOP191	yes	no	yes	yes	yes	4	1199	No
CHOP216	no	no	no	no	yes	2b	983	No
OLHC487	yes	no	no	no	no	2	1694	No
CHOP2199	yes	no	no	no	yes	4	1595	No
OLHC237	yes	no	yes	yes	yes	4	2157	Death
OLHC25	no	no	no	no	no	3	2220	Death

*Death due to disease
